# Endothelial c-Myc knockout disrupts metabolic homeostasis and triggers the development of obesity

**DOI:** 10.3389/fcell.2024.1407097

**Published:** 2024-07-19

**Authors:** Jacqueline F. Machi, Isabella Altilio, Yue Qi, Alejo A. Morales, Diego H. Silvestre, Diana R. Hernandez, Nicolas Da Costa-Santos, Aline G. Santana, Mehrnoosh Neghabi, Parisa Nategh, Thiago L. Castro, João P. Werneck-de-Castro, Mahsa Ranji, Fabiana S. Evangelista, Roberto I. Vazquez-Padron, Ernesto Bernal-Mizrachi, Claudia O. Rodrigues

**Affiliations:** ^1^ Interdisciplinary Stem Cell Institute, Miller School of Medicine, University of Miami, Miami, FL, United States; ^2^ Department of Biomedical Science, Schmidt College of Medicine, Florida Atlantic University, Boca Raton, FL, United States; ^3^ Division of Endocrinology, Diabetes and Metabolism, Department of Internal Medicine, Miller School of Medicine, University of Miami, Miami, FL, United States; ^4^ DeWitt Daughtry Family Department of Surgery, Miller School of Medicine, University of Miami, Miami, FL, United States; ^5^ Department of Electrical Engineering and Computer Science, College of Engineering and Computer Science, Florida Atlantic University, Boca Raton, FL, United States; ^6^ School of Arts, Sciences and Humanities, University of São Paulo, São Paulo, Brazil

**Keywords:** MYC, endothelial dysfunction, adiposity, metabolism, obesity, glucose intolerance

## Abstract

**Introduction:** Obesity is a major risk factor associated with multiple pathological conditions including diabetes and cardiovascular disease. Endothelial dysfunction is an early predictor of obesity. However, little is known regarding how early endothelial changes trigger obesity. In the present work we report a novel endothelial-mediated mechanism essential for regulation of metabolic homeostasis, driven by c-Myc.

**Methods:** We used conditional knockout (EC-Myc KO) and overexpression (EC-Myc OE) mouse models to investigate the endothelial-specific role of c-Myc in metabolic homeostasis during aging and high-fat diet exposure. Body weight and metabolic parameters were collected over time and tissue samples collected at endpoint for biochemical, pathology and RNA-sequencing analysis. Animals exposed to high-fat diet were also evaluated for cardiac dysfunction.

**Results:** In the present study we demonstrate that EC-Myc KO triggers endothelial dysfunction, which precedes progressive increase in body weight during aging, under normal dietary conditions. At endpoint, EC-Myc KO animals showed significant increase in white adipose tissue mass relative to control littermates, which was associated with sex-specific changes in whole body metabolism and increase in systemic leptin. Overexpression of endothelial c-Myc attenuated diet-induced obesity and visceral fat accumulation and prevented the development of glucose intolerance and cardiac dysfunction. Transcriptome analysis of skeletal muscle suggests that the protective effects promoted by endothelial c-Myc overexpression are associated with the expression of genes known to increase weight loss, energy expenditure and glucose tolerance.

**Conclusion:** Our results show a novel important role for endothelial c-Myc in regulating metabolic homeostasis and suggests its potential targeting in preventing obesity and associated complications such as diabetes type-2 and cardiovascular dysfunction.

## 1 Introduction

Overweight and obesity are chronic conditions that result mostly from exposure to hypercaloric diet rich in saturated fat and sugar and lack of physical activity (Faruque et al., 2019; Elmaleh-Sachs et al., 2023). Other factors such as aging, stress, certain health conditions, medication, and genetics have also been associated with the development of overweight and obesity. Obese individuals have an increased risk of developing cardiovascular disease, liver disease, diabetes, and cancer (Scully et al., 2020; Powell-Wiley et al., 2021).

Endothelial cells play an essential role in tissue homeostasis by supporting regeneration through the regulation of surveillance and repair mechanisms (Reiterer and Branco, 2020). Endothelial dysfunction, which occurs with aging and exposure to environmental stress factors, has a detrimental impact on organ physiology, ultimately leading to multiple pathological conditions such as obesity, type-2 diabetes, and cardiovascular disease (Engin, 2017; Donato et al., 2018; Haybar et al., 2019; Kajikawa and Higashi, 2022). Most studies on obesity have focused on how exposure to a diet rich in fat triggers endothelial dysfunction. However, little is known regarding how early changes in the endothelium contribute to obesity.

The transcription factor c-Myc plays an important physiological role controlling multiple cellular functions (Hofmann et al., 2015; Zacarias-Fluck et al., 2024). Deregulated c-Myc expression has been associated with cancer, metabolic and inflammatory conditions (Zheng et al., 2017; Luo et al., 2021; Rosselot et al., 2021; Nevzorova and Cubero, 2023; Zacarias-Fluck et al., 2024). Several reports from our group and others have highlighted an essential role of the transcription factor c-Myc in endothelial cell function in development (Baudino et al., 2002; He et al., 2008; Rodrigues et al., 2008; Kokai et al., 2009) and disease (Riu et al., 2002; Riu et al., 2003; Hurley et al., 2010; Florea et al., 2013; Rosselot et al., 2019; Qi et al., 2022). In addition to regulating angiogenesis, we have shown that c-Myc controls endothelial self-renewal and inflammation (Florea et al., 2013; Qi et al., 2022). In the present work, we provide supporting evidence that endothelial c-Myc plays an essential role in regulating overweight and obesity, which extends to the prevention of insulin resistance and cardiovascular dysfunction.

## 2 Materials and methods

### 2.1 Animals

All animal experiments were approved by the University of Miami Animal Care and Use Committee according to the National Institutes of Health guidelines and conform to the Guide for the Care and Use of Laboratory Animals. A total of four transgenic mouse lines were used in this study to conditionally regulate c-Myc expression in endothelial cells. All animals were housed on a 12-h light/dark cycle with free access to food and water unless otherwise stated. Endothelial c-Myc knockout mice were generated by crossing c-Myc^*flox/flox*^ (B6.129S6-*Myc* ^*tm2Fwa*^/Mmax, Strain #032046, Jackson Laboratory, Bar Harbor, ME, USA) and Cdh5(PAC)-CreERT2 (C57BL/6-Tg (Cdh5-cre/ERT2)1Rha), developed by Dr. Ralph Adams at Cancer Research UK (Cancertools.org reference number 151520) mouse lines. Knockout controls consisted of littermates carrying identical floxed genotypes, but lacking Cre (Cre-negative) or carrying only the Cre (Flox/Flox-negative). Males and females were used in the study. Induction of c-Myc knockout was performed between 4-6 weeks of age through daily intraperitoneal injections of 2 mg tamoxifen (#13258, Cayman Chemical, Ann Arbor, MI, USA) per animal for a total period of 5 days as previously described (Qi et al., 2022). Endothelial c-Myc overexpression mice were generated by crossing Tet-O-Myc (FVB/N-Tg(tetO-MYC)36aBop/J, Stanford University, Stanford, CA, USA) and Cdh5-tTA (FVB-Tg(Cdh5-tTA)D5Lbjn/J, Strain #013585 Jackson Laboratory, Bar Harbor, ME, USA) mouse lines in the presence of doxycycline diet (#TD.01306, Teklad, Indianapolis, IN, USA) to prevent the expression of the human c-Myc transgene. Overexpression controls consisted of littermates carrying the Tet-O-Myc genotype but lacking the transactivator (tTA-negative). Induction of c-Myc overexpression was performed between 4-6 weeks of age through withdrawal from the doxycycline diet. Only males were used in the study because the human c-Myc transgene is restricted to the Y-chromosome in this model. c-Myc knockout and overexpression in endothelial cells were confirmed by qPCR (Supplementary Figure S1).

For diet-induced obesity experiments, mice were exposed to control (#TD.08485) or high-fat diet (#TD.88137) (Teklad, Indianapolis, IN, USA) for a total period of 20 weeks.

### 2.2 Vasoactive response studies

Vasoactive response was assessed in mesenteric arteries harvested from control (CT) and endothelial c-Myc knockout (EC-Myc KO) mice using a pressure myograph system model 110p (Danish Myo Technology, Denmark) as previously described (Hernandez et al., 2019). Briefly, the mesenteric arcade was initially isolated and placed in physiological salt solution (PSS) for dissection of the second-order mesenteric arteries and preserve viability. Vessels were then mounted on two glass microcannulas and placed in the myograph chamber containing PSS solution at 37°C and under aeration with a special mix of 95% O_2_ and 5% CO_2_. Quality control was performed to confirm vessel viability prior to all measurements as previously described (Coats and Hillier, 1999). After confirmation of viability, arteries were washed with PSS, and pre-contracted with norepinephrine followed by assessment of endothelium-dependent vasodilation through exposure to increasing doses of acetylcholine every 5 min. Pressure-outside diameter curves were recorded at an isobaric condition of 60 mmHg as evidence of vasoreactivity response for different acetylcholine concentrations.

### 2.3 Gross phenotypic analysis

We performed longitudinal analysis of experimental animals maintained under a standard control or a western style high-fat diet. Body weight was collected once a week (high-fat diet) or once a month (aging), and animals were macroscopically evaluated for any visible abnormalities. Food consumption was monitored in EC-Myc KO animals. Any signs of abnormalities, cancer development and sudden deaths were recorded.

### 2.4 Indirect calorimetry and body composition measurements

Whole-body energy metabolism was evaluated in CT and EC-Myc KO mice by indirect calorimetry using the Oxymax Comprehensive Lab Animal Monitoring System (CLAMS) (Columbus Instruments, Columbus, OH, USA) as previously described (Fonseca et al., 2014). Chambers were maintained at 22°C and O_2_ levels were calibrated against a standard gas mixture prior to use. Mice were individually housed under light/dark cycles of 12-h and allowed to acclimate to the chamber for 48 h prior to data collection. Food and water were available *ad libitum*. After acclimation, O_2_ consumption, CO_2_ production, respiratory exchange ratio (RER) and heat production were collected at 26-min intervals for a total period of 48 h.

### 2.5 Metabolic assessment of skeletal muscle slices

The redox state of skeletal muscle tissue harvested from CT and EC-Myc KO mice was estimated using a 3D fluorescence cryo-imaging system custom designed by the Biophotonics Laboratory at Florida Atlantic University (Ceyhan et al., 2023). Briefly, frozen tissues were embedded a day before imaging in a black absorbent medium. For imaging, the frozen tissue block was mounted to the sample stage, where its temperature was maintained at cryogenic temperatures (−10°C) for a higher quantum yield of fluorescence while retaining markers of metabolic state. A motor-driven stage and microtome blade allowed tissue slicing at 30 µm thickness. Images were acquired using a CCD camera (Retiga R6, Teledyne Photometrics, Tucson, AZ) with alternating filter wheels for nicotinamide adenine dinucleotide (NADH), and oxidized flavin adenine dinucleotide (FAD). The tissue was excited with a mercury arc lamp (200W lamp, Oriel, Irvine, CA). The broad light passes through excitation filters 350 ± 40 nm (UV Pass Blacklite, HD Dichroic, Los Angeles, CA) for the NADH channel and 437 ± 10 nm (440QV21, Omega Optical, Brattleboro, VT) for the FAD channel. All components of image acquisition operate with an automated virtual interface LabVIEW software (2022, National Instruments). The images of each slice were stacked in the *z*-direction to generate 3D images. Variables such as light intensity, illumination pattern, and dark current noise were considered for image processing. The redox ratio (RR = NADH/FAD) was calculated by dividing the fluorescence values of NADH over FAD images voxel by voxel. Representative images of NADH and FAD intensity are provided in Supplementary Figure S2.

### 2.6 Glucose tolerance test

Mice were fasted for 6-h with continuous access to water before the glucose load (i.p. bolus of 1.5 mg/kg body weight). Glucose levels were determined from a small drop of blood collected from the tail using a commercially available glucometer (AlphaTRAK^®^, Zoetis, United Kingdom). Samples were collected before and 15, 30, 60, 90 and 120 min after glucose administration.

### 2.7 Pathology and biochemical analysis

At endpoint, blood and major organs were collected for pathology analysis from all experimental groups. Tissues were macroscopically examined for visible signs of disease and organ weight was recorded. Blood was collected by cardiac puncture from fasted (approximately 6-h) and non-fasted animals. Plasma and serum were separated from blood for analysis of insulin and leptin levels using commercially available ELISA kits (#EZRMI and #RAB0334, Sigma-Aldrich Inc., St Louis, MO) per manufacture instructions.

### 2.8 Endothelial cell sorting and c-Myc expression analysis

Endothelial cells were magnetically sorted from hearts harvested from CT and EC-Myc OE mice based on CD31 expression using commercially available kits and instrument (Miltenyi Biotec Inc., Gaithersburg, MD). Briefly, harvested hearts were minced and dissociated with a mixture of enzymes (Multi Tissue Dissociation Kit 2, #130-110-203) using the gentleMACS Octo Dissociator system. After dissociation, cell suspension was cleared of debris (Debris Removal Solution, #130-109-398) and incubated with CD45 microbeads (#130-052-301) to exclude inflammatory cells, which may also express CD31. The CD45 depleted cell suspension was then incubated with CD31 microbeads (#130-097-418) for final sorting of endothelial cells. All steps followed manufacturer’s instructions for each specific kit used. RNA was extracted from sorted endothelial cells for analysis of mouse and human c-Myc by qPCR using Taqman probes (#Mm00487804_m1 and #Hs99999003_m1, respectively) per manufacture instructions (Life Technologies Corp, Carlsbad, CA).

### 2.9 Gene expression analysis by RNA-sequencing

RNA was extracted from soleus and gastrocnemius muscle harvested from CT and EC-Myc OE mice using TRI-reagent (#TR118, Molecular Resource Center Inc., Cincinnati, OH) per manufacturer’s instructions. RNA concentration was determined using a NanoDrop spectrophotometer (Life Technologies Corp, Grand Island, NY) and samples were outsourced for library preparation, sequencing, and bioinformatics analysis (Novogene Corporation, Inc. Sacramento, CA). Prior to library preparation, RNA integrity was evaluated using an Agilent BioAnalyzer 2100 (Agilent Technologies), and messenger RNA was purified from total RNA using poly-T oligo-attached magnetic beads. The library was checked with Qubit and real-time PCR for quantification and bioanalyzer for size distribution detection.

### 2.10 Statistical analysis

We used SigmaPlot (Inpixon) and Prism (GraphPad) Software for all graphs and statistical analysis. Sample numbers are indicated in all figure legends and *p*-values <0.05 were considered significant. For comparison between two groups, we performed Student’s t*-*test and considered two-tailed *p*-values. When samples did not pass the normality and variance tests, Welch’s *t*-test was used as an alternative. For comparison between four experimental groups, we performed two-way ANOVA. Holm-Sidak test was used for multiple comparison analysis. Tukey HSD tests were performed as a *post hoc* test to identify significant differences between individual groups. Two-way RM ANOVA was used for the interaction effect of the genotype with one continuous dependent variable. Individual animals are represented by dots in all graphs. Bars indicate the standard deviation unless otherwise stated.

## 3 Results

### 3.1 Endothelial c-Myc knockout impairs vasorelaxation

**FIGURE 1:**
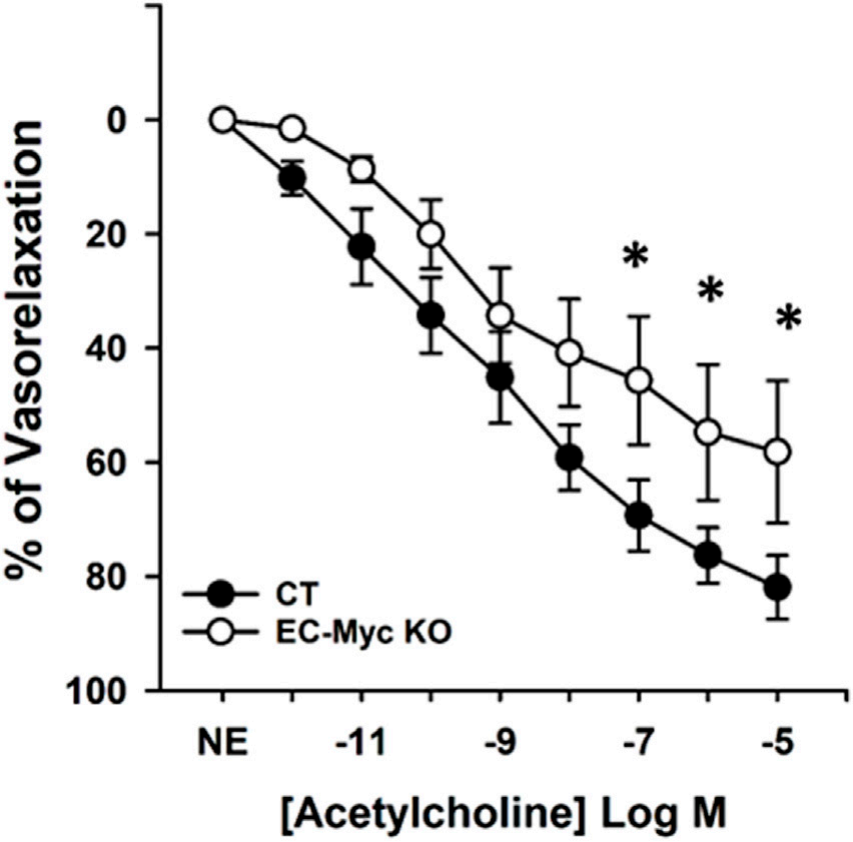
Vasoactive response of mesenteric artery from control and endothelial c-Myc knockout mice. Curves indicate vasorelaxation response to acetylcholine 1 month after induction of endothelial c-Myc knockout in male mice. Results are expressed as percentage of vasorelaxation and represent the mean ± standard error for individual concentrations of acetylcholine. Black and white circles represent control and knockout mice, respectively. CT, Control (*n* = 4); EC-Myc KO, Endothelial c-Myc Knockout (*n* = 4); NE, Norepinephrine. *p < 0.05.

Endothelial dysfunction is an early predictor of multiple pathological conditions. Little is known regarding early mechanisms that trigger endothelial dysfunction. Previous studies from our group have shown that the transcription factor c-Myc plays an important role in endothelial homeostasis and regulation of inflammation (Florea et al., 2013; Qi et al., 2022). These findings propelled us to investigate if c-Myc contributes to endothelial dysfunction ultimately impacting overall animal health. To confirm the association between c-Myc deficiency and endothelial dysfunction, we performed vasoactive response studies as impaired vasorelaxation is one of the first signs of dysfunctional endothelium. Mesenteric arteries of control (CT) and endothelial c-Myc knockout (EC-Myc KO) mice were harvested and tested for acetylcholine-induced vasorelaxation after constriction with norepinephrin. Our results show that loss of endothelial c-Myc significantly impacted vasorelaxation in response to acetylcholine relative to control. At the highest dose of acetylcholine tested (10^−5^ M), the ability of EC-Myc KO mesenteric arteries to relax was reduced by 29% relative to CT mice (58.2% ± 12.5% vs. 81.9% ± 5.6%, *p* = 0.031) (Figure 1).

### 3.2 Endothelial c-Myc knockout increases body weight and adiposity

**FIGURE 2:**
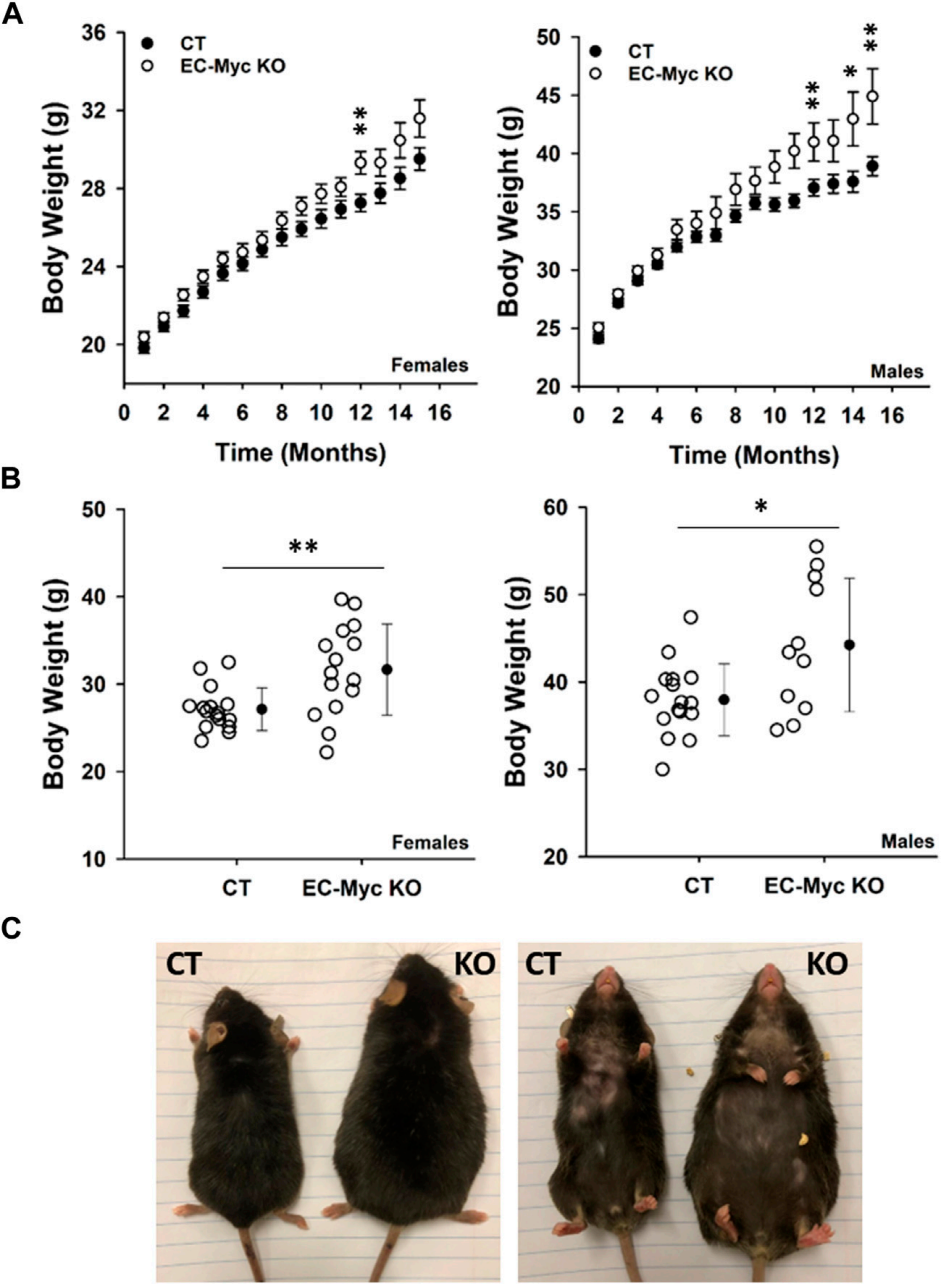
Body weight analysis of control and endothelial c-Myc knockout mice during aging. **(A)** Longitudinal analysis of body weight in male and female mice. Black and white circles represent control and knockout mice, respectively. Results represent the mean ± standard error **(B)** Analysis of body weight at 16-month endpoint. Dots represent individual animals and filled circles represent the mean ± standard deviation. **(C)** Representative dorsal and ventral images of males showing size differences between experimental groups. CT, Control (*n* = 19–65); EC-Myc KO, Endothelial c-Myc Knockout (*n* = 17–49). *p < 0.02, **p < 0.002.

We performed longitudinal analysis of CT and EC-Myc KO mice over a period of 16 months and found an expected increase in body weight over time, although findings were more pronounced in knockout animals relative to control (Figure 2A). At endpoint, body weight was significantly increased in EC-Myc KO females (31.67 ± 1.35 vs. 27.13 ± 0.61 g, *p* = 0.004) and males (44.2 ± 2.30 vs. 38.0 ± 1.03 g, *p* = 0.01) relative to CT (Figure 2B). When compared side by side, EC-Myc KO mice looked bigger in size than CT (Figure 2C).

**FIGURE 3:**
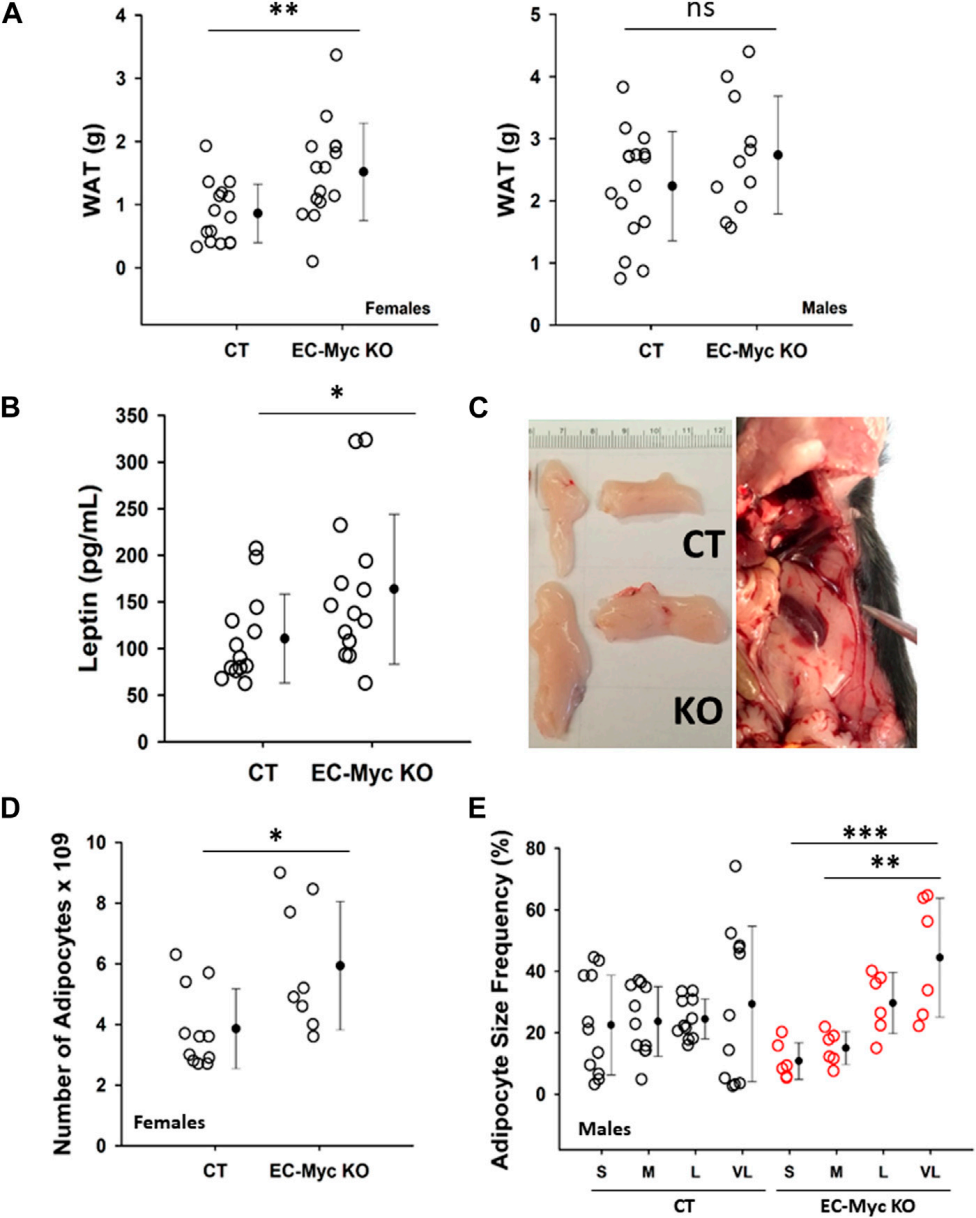
Analysis of white adipose tissue in control and endothelial c-Myc knockout mice. **(A)** Quantification of white adipose tissue mass. **(B)** Quantification of leptin levels. **(C)** Representative images of fat deposits. The image on the right corresponds to an extreme case of increased adiposity in knockout mice. **(D)** Morphometric analysis of white adipose tissue showing an increase in the number of adipocytes in female knockout mice. **(E)** Morphometric analysis of white adipose tissue showing significant difference in adipocyte size distribution in male knockout mice. Dots represent individual animals and filled circles represent the mean ± standard deviation. CT, Control (*n* = 10–16); EC-Myc KO, Endothelial c-Myc knockout (*n* = 5–15); S, small; M, medium; L, large; VL, very large; ns, non-significant. *p < 0.05, **p < 0.01, ***p < 0.005.

Analysis of white adipose tissue (WAT) at endpoint in EC-Myc KO mice showed a significant increase by 76% in females (1.52 ± 0.20 vs. 0.86 ± 0.12, *p* = 0.007) and a trend increase by approximately 22% in males (2.74 ± 0.29 vs. 2.24 ± 0.22 g, not significant) (Figure 3A). Based on the observed increase in white adipose tissue (WAT) accumulation, in endothelial c-Myc deficient animals, we analyzed the circulating levels of leptin, which is expected to correlate with changes in adipocyte mass (Kiernan and MacIver, 2020). Our results showed a significant increase of approximately 47.98% in serum leptin in EC-Myc KO animals relative to CT (163.72 ± 21.48 vs. 110.64 ± 13.17 pg/mL) (Figure 3B). Representative images of fat deposits from CT and EC-Myc KO mice are shown in Figure 3C. Morphometric analysis of WAT from male and female EC-Myc KO animals revealed some sex-specific differences relative to CT (Supplementary Table S1). In EC-Myc KO females, we found a significant increase of 54% in the estimated number of adipocytes relative to CT (5.93 ± 0.75 vs. 3.85 ± 0.40, *p* = 0.025), but no changes in other parameters (Figure 3D). In males, the only significant difference between CT and EC-Myc KO was in the frequency of adipocyte size. EC-Myc KO males showed a higher percentage of very large-sized adipocytes relative to small sizes, while no differences were found in CT (Figure 3E).

### 3.3 Endothelial c-Myc knockout reduces metabolic activity

**FIGURE 4:**
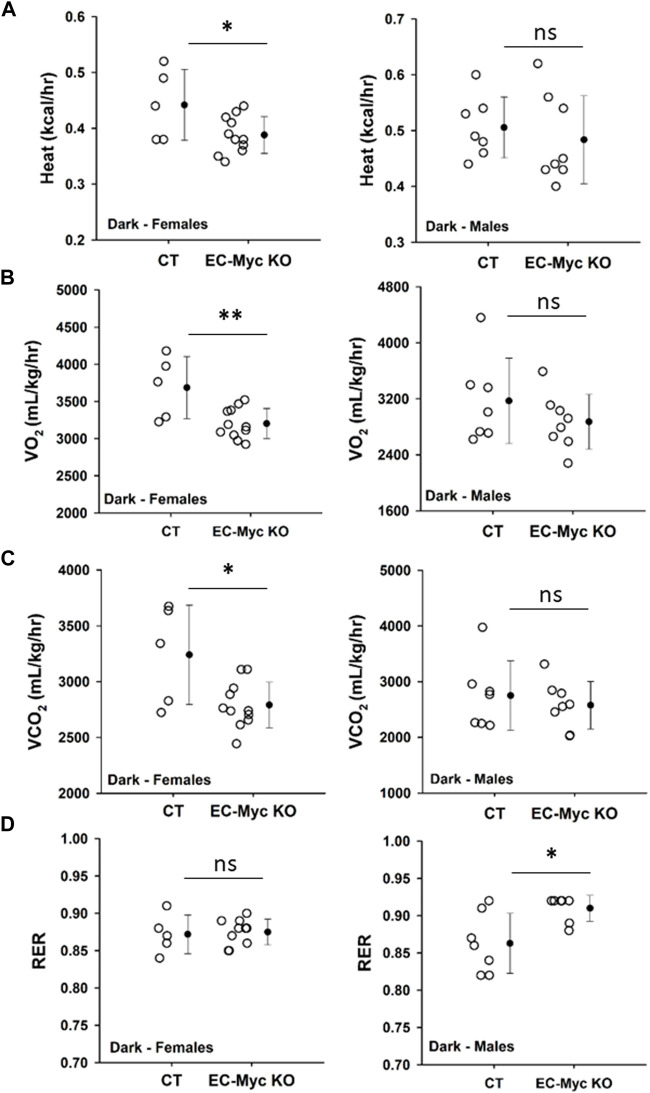
Metabolic phenotype of control and endothelial c-Myc knockout mice by indirect calorimetry. **(A)** Heat production. **(B)** Volume of oxygen consumed (VO_2_). **(C)** Volume of carbon dioxide release (VCO_2_). **(D)** Respiratory exchange ratio (RER). Dots represent individual animals and filled circles represent the mean ± standard deviation. CT, Control (*n* = 5–7); EC-Myc KO, Endothelial c-Myc Knockout (*n* = 7–11); ns, non-significant. *p < 0.05, **p < 0.01.

Our observations described above suggest that loss of endothelial c-Myc causes an imbalance in energy metabolism. We analyzed food intake in CT and EC-Myc KO mice prior to the onset of weight gain and did not find any significant differences (data not shown). In the absence of alterations in calorie intake, one possible explanation for the observed increase in body weight in EC-Myc KO mice is a decrease in energy expenditure. Accordingly, we performed a series of metabolic studies with CT and EC-Myc KO mice by indirect calorimetry and found interesting sex-related differences between experimental groups. Similar results were found in dark and light periods (Supplementary Figure S3). A summary of all metabolic parameters is presented in Supplementary Table S2. In males, we found a significant increase in respiratory exchange ratio in EC-Myc KO relative to CT (0.91 ± 0.01 vs. 0.86 ± 0.02 RER, *p* = 0.015), without significant changes in other metabolic parameters (Figure 4D). However, in females, we found a significant decrease in heat production (0.39 ± 0.01 vs. 0.44 ± 0.03 kcal/kg/h, dark period *p* = 0.04) (Figure 4A), which was accompanied by a decrease in VO_2_ (3203 ± 61 vs. 3687 ± 187 mL/kg/h, *p* = 0.007) (Figure 4B) and CO_2_ (2791 ± 62 vs. 3241 ± 199 mL/kg/h, *p* = 0.01) (Figure 4C). No significant differences in RER were found in females (Figure 4D).

**FIGURE 5:**
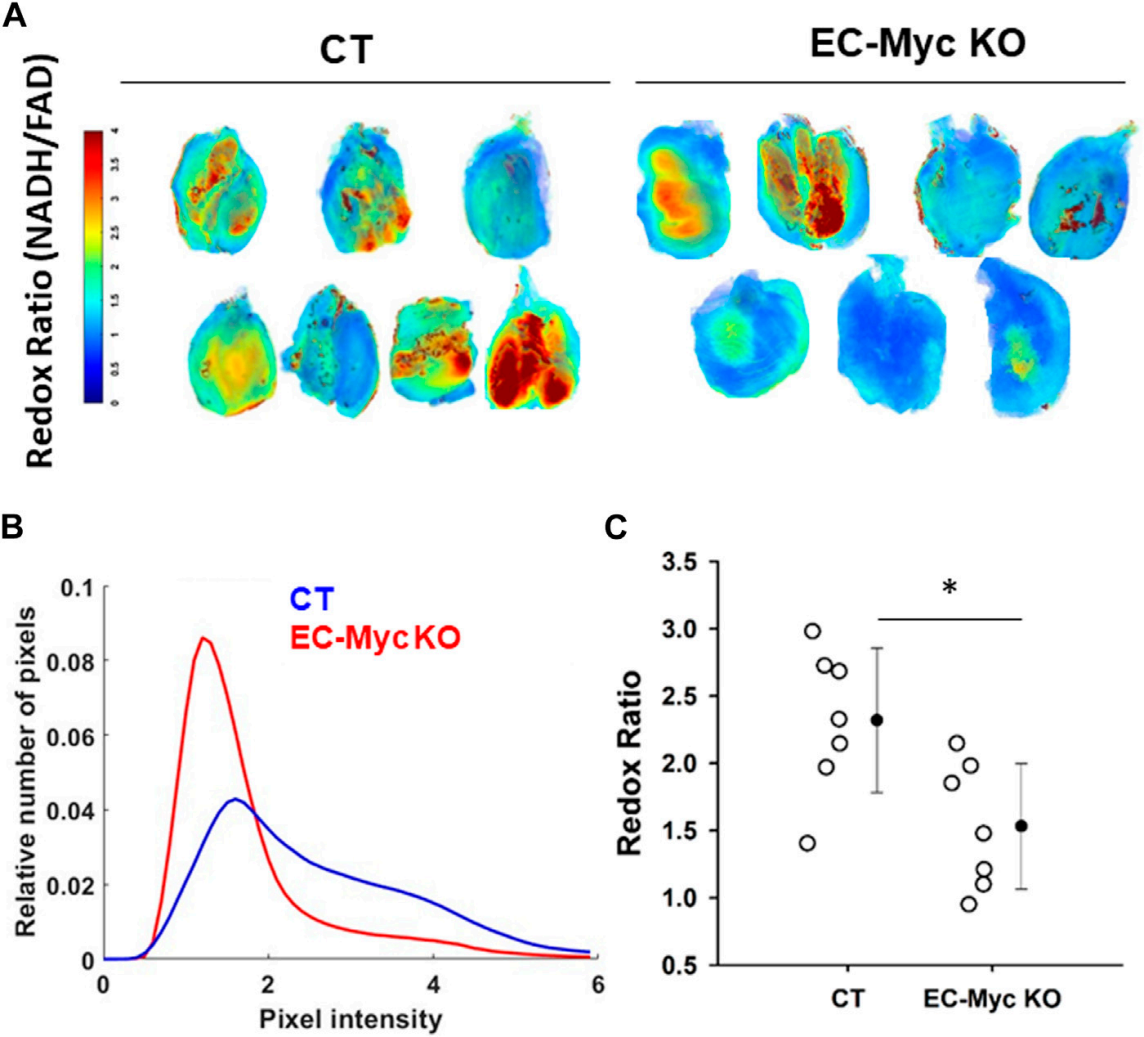
Analysis of redox ratio in control and endothelial c-Myc knockout skeletal muscle. **(A)** Normalized intensities of redox ratio. For each group, seven representative muscle images are presented. **(B)** Histogram plot showing decrease in redox ratio in skeletal muscle of endothelial c-Myc knockout mice relative to control. **(C)** Average redox ratio of individual animals. Dots represent individual animals and filled circles represent the mean ± standard deviation. CT, Control (*n* = 7); EC-Myc KO, Endothelial c-Myc knockout (n = 7). *p < 0.05.

To further explore the impact of endothelial c-Myc loss in energy metabolism, we performed analysis of redox ratio in skeletal muscle harvested from CT and EC-Myc KO animals. Our results indicated a significant reduction of 51% in the mitochondrial redox ratio in EC-Myc KO relative to CT animals (1.53 ± 0.17 vs. 2.32 ± 0.2), which suggests a decrease in cellular energy production (Figure 5).

### 3.4 Overexpression of c-Myc in endothelial cells attenuates visceral fat accumulation and prevents systemic leptin release induced by western diet exposure

**FIGURE 6:**
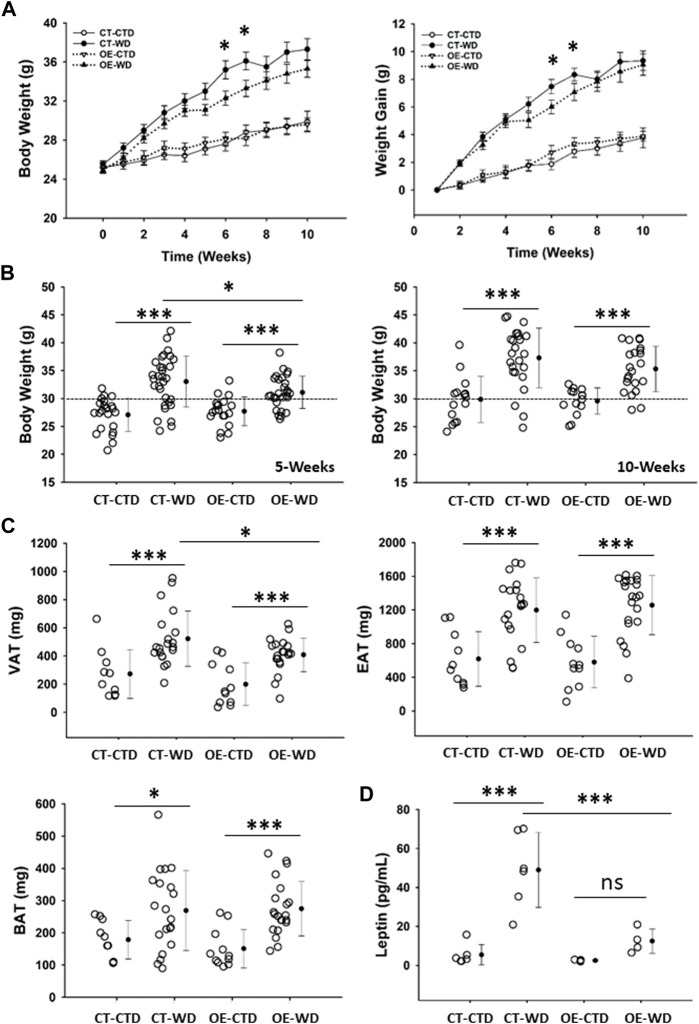
Gross phenotype analysis of control and endothelial c-Myc overexpression mice under exposure to western diet. **(A)** Longitudinal analysis of body weight and weight gain for a total period of 10 weeks. White and black symbols represent animals exposed to control and western diet, respectively. **(B)** Analysis of body weight at 5- and 10-weeks endpoints. **(C)** Quantification of visceral (VAT), epididymal (EAT) and brown (BAT) adipose tissue mass at 10-weeks endpoint. **(D)** Quantification of systemic leptin levels at 10-weeks endpoint. In A, results are represented as mean ± standard error. In all other graphs, dots represent individual animals, filled circles represent the mean ± standard deviation. CT, Control; OE, Endothelial c-Myc overexpression; CTD, control diet (*n* = 14–22); WD, western diet (*n* = 22–31); ns, non-significant. (*p < 0.05, ***p < 0.001).

Our results described above indicate an important role for endothelial c-Myc in the maintenance of metabolic homeostasis. As such, we hypothesized that overexpression of c-Myc in endothelial cells would protect animals from developing overweight and obesity, as well as associated complications such as glucose intolerance and cardiovascular disease. To test this hypothesis, we performed a series of experiments in which we challenged control (CT) and endothelial c-Myc overexpression (EC-Myc OE) mice with a western-style high-fat diet (WD) over a period of 20 weeks. Exposure to WD promoted a gradual increase in body weight in both CT and EC-Myc OE mice (Figure 6A). This effect was significantly attenuated around 5 weeks post-exposure in EC-Myc OE relative to CT (31.10 ± 0.55 vs. 33.03 ± 0.83 g, *p* = 0.035) (Figure 6B). At 10 weeks post-exposure, the difference in body weight between both groups was lost. However, analysis of fat deposits revealed significant attenuation by 22% in visceral adipose tissue accumulation in EC-Myc OE mice relative to CT (408 ± 26.3 vs. 522 ± 43.9 mg, *p* = 0.028) (Figure 6C). No significant differences were observed in epidydimal and brown adipose tissue.

Quantification of systemic leptin levels showed a significant increase in CT animals under WD exposure relative to the control diet (48.98 ± 7.85 vs. 5.39 ± 2.12 pg/mL, *p* < 0.001), while the response was prevented in EC-Myc OE mice (12.46 ± 3.13 vs. 2.47 ± 0.23 pg/mL, not significant). Our findings revealed significant differences between EC-Myc OE and CT under WD exposure (12.46 ± 3.13 vs. 48.98 ± 7.85 pg/mL, *p* < 0.001) (Figure 6D).

### 3.5 Overexpression of c-Myc in endothelial cells prevents the development of western diet-induced glucose intolerance

**FIGURE 7:**
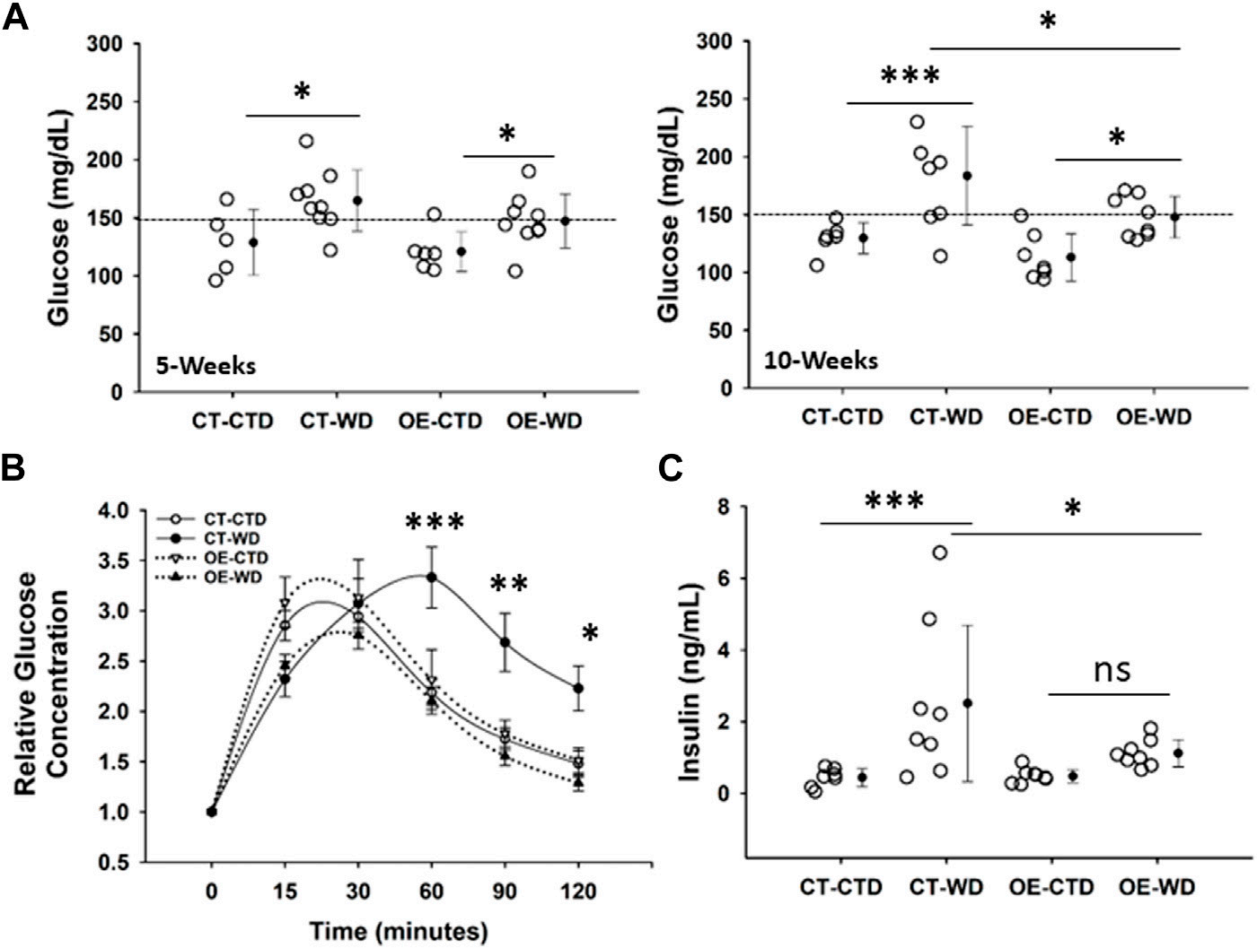
Analysis of glucose tolerance in control and endothelial c-Myc overexpression mice under exposure to western diet. **(A)** Basal glucose levels at 5- and 10-weeks. **(B)** Glucose tolerance test at 10-weeks. White and black symbols represent animals exposed to control and western diet, respectively. **(C)** Quantification of systemic insulin levels at 10-weeks endpoint. In B, results are expressed as fold change relative to baseline and represent the mean ± standard error. In all other graphs, dots represent individual animals and filled circles represent the mean ± standard deviation. CT, Control; OE, Endothelial c-Myc overexpression; CTD, control diet (*n* = 6–7); WD, western diet (8-9). *p < 0.05, **p < 0.005, ***p < 0.001.

One of the major complications associated with obesity is the development of insulin resistance. CT and EC-Myc OE mice have similar fasting glucose levels under normal diet conditions. Exposure to WD for 5 weeks was sufficient to significantly raise basal glucose levels in both CT (164 ± 8 vs. 129 ± 13 mg/dL, *p* = 0.013) and EC-Myc OE (147 ± 8 vs. 121 ± 7 mg/dL, *p* = 0.048) mice relative to the control diet. However, after 10-weeks of exposure to WD, although basal glucose further increased in CT animals relative to control diet (184 ± 15 vs. 130 ± 5 mg/dL, *p* = 0.001), it remained the same in EC-Myc OE mice (148 ± 6 vs. 113 ± 8 mg/dL, *p* = 0.02). Our findings indicated significant differences in basal glucose between EC-Myc OE and CT mice in response to long-term exposure to WD (148 ± 6 vs. 184 ± 15 mg/dL, *p* = 0.014) (Figure 7A).

We performed a glucose tolerance test (GTT) in all experimental groups to account for the development of glucose intolerance. Under the WD diet, the time to glucose peak in CT was longer than in EC-Myc OE (60 vs. 30 min). After 120 min, the level of glucose in EC-Myc OE mice under WD was almost completely back to baseline (211.00 ± 92.25 vs. 161.11 ± 43.44 mg/dL, 30% above baseline), while in CT animals, it remained significantly elevated (402.88 ± 121.50 vs. 183.83 ± 42.64 mg/dL, 120% above baseline) (Figure 7B).

At the 10-weeks endpoint, we measured systemic insulin levels and found a significant increase in CT animals exposed to WD relative to control diet (2.51 ± 2.18 vs. 0.44 ± 0.26 ng/mL, *p* = 0.001), which was significantly attenuated in EC-Myc OE animals (1.12 ± 0.38 vs. 0.48 ± 0.18 ng/mL, not significant). Our findings indicated significant differences in insulin levels between EC-Myc OE and CT mice in response to WD (1.12 ± 0.13 vs. 2.51 ± 0.77 ng/mL, *p* = 0.019) (Figure 7C).

### 3.6 Transcriptome analysis of skeletal muscle revealed significant differences between control and endothelial c-Myc overexpression mice in response to western diet exposure

The skeletal muscle plays an important role in energy metabolism (Mengeste et al., 2021). Based on our findings suggesting that endothelial c-Myc overexpression attenuates visceral fat accumulation and insulin resistance, we performed transcriptome analysis of skeletal muscle harvested from CT and EC-Myc OE mice. Exposure to WD for 10 weeks had a significant impact on the gene expression profile of both CT (1016 genes altered >1.5-fold) and EC-Myc OE mice (666 genes altered >1.5-fold) relative to control diet. Venn diagram analysis showed that both experimental groups shared common targets altered by diet exposure (243 genes) and that each experimental group had their own exclusive list of altered genes. It was noticeable that the number of genes in CT was almost double of what was found in EC-Myc OE (709 vs. 422 genes) (Supplementary Figure S4).

Comparison of the transcriptome profiles of WD-treated groups showed a total of 207 genes (128-up and 79-down) significantly altered >1.5-fold in EC-Myc OE (OE-WD) relative to CT (CT-WD). Among the most significantly altered targets, we identified *Zbtb16* (2.35-fold, *p* = 1.39 × 10^−19^, *Acsl3* (1.56-fold, *p* = 2.4 × 10^−10^) and *Rabif* (2.04-fold, *p* = 2.36 × 10^−07^) as the top upregulated genes, and *Rrad* (−1.57-fold, *p* = 1.61 × 10^−5^) as the top downregulated. The top canonical pathways identified by Ingenuity software analysis that are affected by endothelial c-Myc expression are shown in Supplementary Figure S5. The S100 Family Signaling was among the top 5, comprising up- and downregulated genes. Some of the targets in this pathway include *S100a3* (6.9-fold, *p* = 0.009, *S100a14* (2.6-fold, *p* = 0.043) and *Wnt10a* (3.96-fold, *p* = 0.002).

**FIGURE 8:**
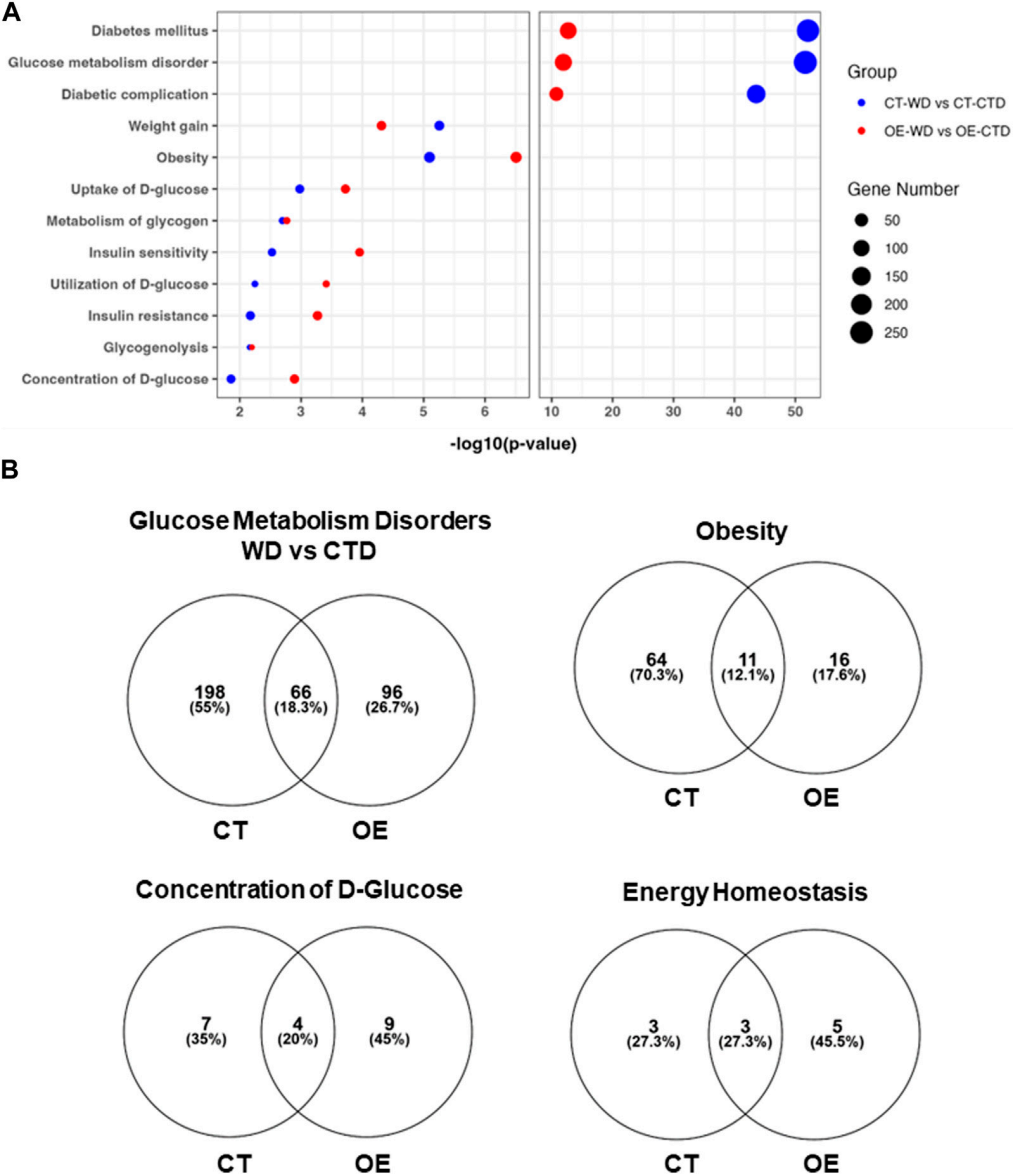
Transcriptome analysis of skeletal muscle from control and endothelial c-Myc overexpression mice. **(A)** Top metabolic-related diseases and functions affected by western diet exposure in control and endothelial c-Myc overexpression mice relative to control diet. **(B)** Venn diagram analysis showing the number of gene targets altered by western diet exposure in each metabolism-related disease or function. CT, Control; OE, Endothelial c-Myc overexpression; CTD, control diet (*n* = 3); WD, western diet (*n* = 4).

We next analyzed the transcriptome profile associated with the exclusive response of EC-Myc OE and CT to WD relative to control diet (CTD). Pathway Analysis of upregulated genes differentially expressed showed significant increase in targets associated with extracellular matrix organization, collagen metabolism and organization for both groups. Analysis of downregulated genes revealed interesting differences between groups, including pathways associated with metabolism (Supplementary Figure S6). We focused our analysis on functions specifically related to metabolic disease based on our physiological and pathological findings. Both experimental groups showed changes in the expression of genes associated with diabetes, obesity, insulin resistance/sensitivity, glucose metabolism disorders, weight gain and energy homeostasis. However, the CT group showed a much higher number of genes altered under these function categories than EC-Myc OE (Figure 8). Interestingly, our analysis revealed other metabolic functions for EC-Myc OE that could account for the beneficial effects we observed. Some of the genes differentially expressed EC-Myc OE muscle have been related to weight loss, energy expenditure and glucose tolerance. Among the genes under these exclusive categories, we identified *Socs3* (−1.83-fold, *p* = 0.009) as a common target in multiple pathways.

### 3.7 Endothelial c-Myc overexpression prevents western diet-induced cardiovascular dysfunction and remodeling

**FIGURE 9:**
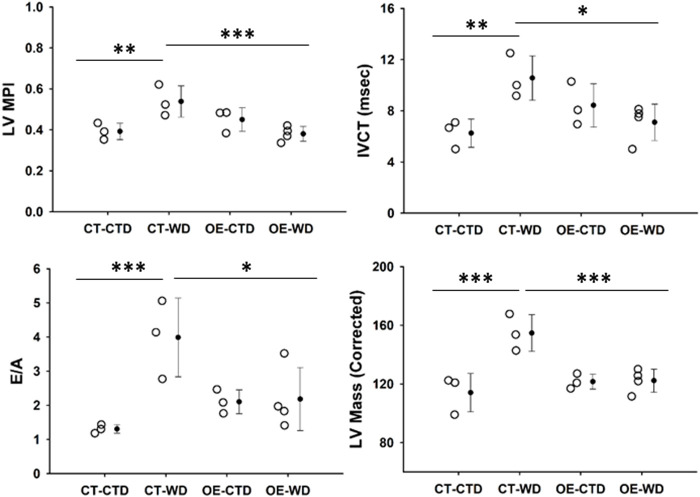
Transthoracic echocardiography analysis of control and endothelial c-Myc overexpression mice. Animals were analyzed after 18-weeks under control and western diet. **(A)** Myocardial performance index (MPI). **(B)** Isovolumetric contraction time (IVCT). **(C)** E/A ratio. **(D)** Left ventricular mass. Dots represent individual animals and filled circles represent the mean ± standard deviation. LV, Left ventricle; CT, Control; OE, Endothelial c-Myc overexpression; CTD, control diet (*n* = 3–4); WD, western diet (*n* = 3–4). *p < 0.05, **p < 0.01, ***p < 0.005.

Obesity is a major risk factor associated with the development of cardiovascular disease. Based on the protective effect of endothelial c-Myc overexpression described above, we performed cardiovascular assessment of animals exposed to control and WD for 18 weeks by echocardiography. It is noticeable from looking at our data that endothelial c-Myc overexpression prevents several functional and structural diet-induced alterations observed in controls. CT animals showed an increase in myocardial performance index (MPI) relative to those fed control diet (0.54 ± 0.08 vs. 0.39 ± 0.04, *p* = 0.008), which was related to an increase in isovolumetric contraction time (IVCT) (10.56 ± 1.73 vs. 6.25 ± 1.10 m, *p* = 0.007). No significant changes in isovolumetric contraction time (IVRT) were observed. In addition, CT mice showed an increase in E/A ratio (3.99 ± 1.15 vs. 1.31 ± 0.13, *p* = 0.02). Interestingly, no significant changes in functional parameters were found in EC-Myc OE mice exposed to WD. At structural level, WD induced a significant increase in CT left ventricular mass (154.71 ± 12.50 vs. 114.12 ± 13.07 mg, *p* < 0.001), whereas no changes were found in EC-Myc OE (Figure 9). However, significant changes in wall thickness were found in both CT and EC-Myc OE mice after exposure to WD. We observed that EC-Myc OE mice under normal diet showed some baseline changes relative to CT, such as an increased fractional shortening (41.82% ± 1.78% vs. 32.64% ± 4.16%, *p* = 0.008), a reduced systolic diameter (1.94 ± 0.15 vs. 2.56 ± 0.39, *p* = 0.025) and increased thickness of the left ventricle posterior wall during systole (1.73 ± 0.18 vs. 1.30 ± 0.28 mm, *p* = 0.014) and diastole (1.33 ± 0.12 vs. 0.91 ± 0.25 mm, *p* = 0.008). A summary of all cardiac parameters is presented in Supplementary Table S3).

## 4 Discussion

In this study, we aimed to elucidate the role of endothelial c-Myc in metabolic homeostasis. Our findings underscore a novel endothelial-mediated mechanism associated with the maintenance of metabolic and cardiovascular health regulated by c-Myc.

Aging and exposure to stress factors have been reported to cause endothelial dysfunction, which is an early predictor of multiple pathological conditions, including obesity, diabetes, and cardiovascular disease (Engin, 2017; Donato et al., 2018; Haybar et al., 2019; Kajikawa and Higashi, 2022). However, recognizing endothelial dysfunction as a cause or an effect in disease conditions needs further investigation. Endothelial cells play a fundamental role in maintaining vascular tone, and impaired vasorelaxation is a primary sign of endothelial dysfunction (Donato et al., 2018). Our results show that depletion of c-Myc in endothelial cells is sufficient to decrease acetylcholine-induced vasorelaxation, supporting previous studies that this transcription factor is essential for maintenance of endothelial function (Baudino et al., 2002; He et al., 2008; Rodrigues et al., 2008; Kokai et al., 2009; Hurley et al., 2010; Florea et al., 2013; Qi et al., 2022).

A key finding of our study is the link between endothelial c-Myc loss and metabolic disturbances. We observed an age-dependent increase in body weight and adiposity with c-Myc depletion from the endothelium that was associated with significant decrease in metabolic parameters supporting a novel essential role for endothelial c-Myc in regulating energy metabolism. Importantly, these findings were backed up by our results on endothelial c-Myc overexpression in the context of obesity, which attenuates visceral fat accumulation and prevents insulin resistance and cardiac dysfunction. The relevance of c-Myc for metabolic homeostasis has been reported, but mostly on non-endothelial cell types. Contrarily to our findings, global c-Myc haploinsufficiency has been related to high metabolic rate without changes in adipose tissue mass relative to controls during aging (Hofmann et al., 2015). Exposure to high-fat diet leads to upregulation of c-Myc expression in adipose tissue and intestines (Liu et al., 2017; Luo et al., 2021). Overexpression of c-Myc in β-cells has been associated with the development of diabetes (Laybutt et al., 2002; Cheung et al., 2010), while reduction in c-Myc expression in intestinal cells was shown to improve high-fat-diet-induced obesity and insulin resistance (Luo et al., 2021). Despite these contradictory findings, which would be difficult to reconcile considering the differences in experimental models used, other work supports a protective role for c-Myc as we observed. Multiple lines of evidence suggest that some increment in c-Myc levels is likely beneficial. Recently, we have shown that loss of c-Myc endothelial cells leads to liver fibrosis (Qi et al., 2022). In hepatocytes, c-Myc is essential to drive proliferation during liver regeneration (Zhang et al., 2018; Wang et al., 2022), and overexpression of c-Myc in the liver has been shown to prevent obesity and insulin resistance (Riu et al., 2002; Riu et al., 2003). In pancreatic β-cells, c-Myc has been shown to promote proliferation as part of an adaptation response to glucose exposure (Puri et al., 2018; Rosselot et al., 2019). Treatment of β-cells the small molecule harmine promotes mitogenesis through a mild increase in c-Myc expression (Wang et al., 2015), suggesting the potential targeting of c-Myc in diabetes to improve insulin production.

Whole body metabolism involves crosstalk between multiple organ systems and endothelial cells serve as the interface of this communication, transmitting signals that will impact tissue response according to environmental cues (Castillo-Armengol et al., 2019; Katagiri, 2023). The most evident effect we observed upon knockout of endothelial c-Myc was an increase in adiposity. Our findings were associated with a raise in leptin levels, which is mostly produced by adipose tissue (Kiernan and MacIver, 2020). Although we observed a significant increase in adiposity in male and female EC-Myc-KO mice relative to CT, we found interesting sex-specific differences in the mechanisms associated with adipose tissue expansion. Sex-related differences in adipose tissue distribution have been reported and seem to play a role in the development of obesity and type-2 diabetes (Tchoukalova et al., 2010a; Gavin and Bessesen, 2020). However, the mechanisms involved are not fully understood. The expansion of adipose tissue can be driven by the formation of new adipocytes (hyperplasia), increase in lipid storage (hypertrophy) and/or reduced lipid breakdown (Li and Spalding, 2022). In EC-Myc KO males, even though we did not observe changes in adipocyte number, we found significant alteration in adipocyte size frequency, with higher accumulation of very large adipocytes, suggesting a hypertrophic mechanism. On the other hand, EC-Myc KO females showed an increase in adipocyte numbers, suggesting a hyperplasia response. These findings are supported by previous studies in humans, indicating that adipose tissue in males involves adipocyte hypertrophy and in females, hyperplasia (Tchoukalova et al., 2008; Tchoukalova et al., 2010b). Our findings suggest a novel endothelial-mediated mechanism driven by c-Myc in adipose tissue morphogenesis.

The skeletal muscle plays an important role in energy metabolism and the crosstalk between endothelial and skeletal muscle cells is essential for energy balance, including insulin-dependent glucose metabolism (Mengeste et al., 2021; Pepe and Albrecht, 2023). One of the most remarkable results from our study was the prevention of glucose intolerance by endothelial c-Myc overexpression in response to high-fat diet exposure. Transcriptome analysis of skeletal muscle provided us with important clues regarding potential mechanisms by which endothelial c-Myc promotes metabolic homeostasis. Pathway analysis of transcriptome data showed that CT and EC-Myc OE share multiple biological and disease functions relevant for metabolic homeostasis, but significant differences were found in the number of genes altered and the identity of specific targets. Importantly, we found an enrichment for weight loss, energy expenditure and glucose tolerance functions exclusively in EC-Myc OE. Among potential downstream targets altered in skeletal muscle of EC-Myc OE that could account for protective results, we found *Socs3*, *Foxo1 and Angptl4*. *Socs3* was common to all the biological and disease functions enriched in our data and downregulated in EC-Myc KO skeletal muscle. Exposure to high-fat diet has been shown to induce SOCS3 expression in skeletal muscle and liver and proposed to act as negative regulator of insulin signaling (Ueki et al., 2004). Skeletal muscle specific deletion of SOCS3 protects mice from insulin resistance induced by high-fat diet exposure (Jorgensen et al., 2013), while overexpression impaired glucose homeostasis (Yang et al., 2012). The expression of *Foxo1* was downregulated in EC-Myc OE muscle. Previous studies have shown that overexpression of FoxO1 in skeletal muscle is associated with insulin resistance and glucose intolerance (Kamei et al., 2004; Teaney and Cyr, 2023). Inhibition of FoxO1 has shown positive effects on glucose homeostasis in experimental models of diabetes (Li et al., 2019; Mao et al., 2021) supporting its potential targeting. ANGPTL4, an adipokine mainly secreted by adipose tissue and liver is involved in lipid metabolism, glucose homeostasis, inflammation and angiogenesis (Xu et al., 2005; Cinkajzlova et al., 2018). Increase in *Angptl4* expression has been reported in skeletal muscle and associated with exercise and exposure to fatty acids, where it may be part of an adaptive response to physical activity (Staiger et al., 2009; Raschke and Eckel, 2013; Sabaratnam et al., 2018). We found the *Angptl4* expression was downregulated in EC-Myc OE muscle. This finding aligns with previous studies where genetic inactivation of ANGPTL4 was associated with improved insulin sensitivity and reduced risk of Type 2 diabetes (Gusarova et al., 2018).

## 5 Conclusion

Outside the domain of cancer, c-Myc plays a significant physiological and pathological role (Rosselot et al., 2021; Nevzorova and Cubero, 2023; Prochownik and Wang, 2023; Zacarias-Fluck et al., 2024). Although therapeutic interventions involving direct c-Myc manipulation may present challenges, the protective effects observed across various parameters suggest that its targeting may offer an approach to mitigate obesity-associated complications. Further mechanistic studies are necessary to unravel the precise molecular pathways by which c-Myc exerts its protective effects in whole body metabolism to establish its translational potential. By understanding the downstream targets of c-Myc and identifying key pathways influenced by its activity, we can advance novel strategies to counteract obesity-related complications and improve overall metabolic and cardiovascular health.

## Data Availability

The RNA-Seq data presented in the study are deposited in the Gene Expression Omnibus (GEO) repository under accession number GSE267493.
