# Supplementary material for: Endothelial c-Myc knockout disrupts metabolic homeostasis and triggers the development of obesity
Source: Front Cell Dev Biol. 2024 Jul 19;12:1407097. doi: 10.3389/fcell.2024.1407097 (PMC11294153; doi:10.3389/fcell.2024.1407097)
Supplement: Supplementary file 1 [file DataSheet1.pdf]

Supplementary Table 1 – Morphometric analysis of white adipose tissue harvested from control and endothelial c-Myc mice

	Females		Males	
**Parameters**	**CT**	**EC-Myc KO**	**CT**	**EC-Myc KO**
Area (µm²)	44.19 ± 4.67	47.22 ± 5.48	54.45 ± 6.78	70.18 ± 7.78
Diameter (µm)	7.17 ± 0.40	7.28 ± 0.48	7.88 ± 0.50	9.04 ± 0.49
Volume (pL)	0.25 ± 0.03	0.28 ± 0.04	0.34 ± 0.06	0.49 ± 0.08
Mass (µg) $x10^4$	2.3 ± 0.3	2.6 ± 0.4	3.1 ± 0.5	4.5 ± 0.7
Adipocyte number $x10^9$	3.8 ± 0.4	**5.93 ± 0.8***	6.2 ± 3.3	6.9 ± 3.5
**Adipocyte Size Frequency**	**CT**	**EC-Myc KO**	**CT**	**EC-Myc KO**
Small	16.59 ± 4.34	20.34 ± 6.37	22.51 ± 4.89	10.81 ± 2.43
Medium	21.59 ± 3.23	17.65 ± 2.57	23.66 ± 3.42	15.00 ± 2.20
Large	27.61 ± 2.25	24.81 ± 2.82	24.47 ± 1.97	29.66 ± 4.07
Very Large	34.21 ± 6.82	37.18 ± 6.95	29.36 ± 7.62	**44.45 ± 7.91$^{¥\#}$**

Results represent mean ± standard error. Significant findings are labeled in bold. **CT**, Control (n = 10-12); **EC-Myc KO**, Endothelial c-Myc knockout (n = 5-8). *p<0.05 EC-Myc KO vs CT (females), $^{¥}$p<0.01 very large vs medium size particles, $^{\#}$p<0.005 very large vs small size particles in EC-Myc KO (males).

Supplementary Table 2 – Metabolic assessment of control and endothelial c-Myc knockout mice

	Females		Males	
	**CT**	**EC-Myc KO**	**CT**	**EC-Myc KO**
VO_2 (ml/kg/h) Dark	3687 ± 187	**3203 ± 61***	3170 ± 232	2871 ± 139
VO_2 (ml/kg/h) Light	3035 ± 163	**2551 ± 84***	2450 ± 187	2235 ± 129
VCO_2 (ml/kg/h) Dark	3240.8 ± 199	**2791 ± 62***	2750 ± 235	2576 ± 151
VCO_2 (ml/kg/h) Light	2557 ± 157	**2146 ± 94***	2063 ± 182	1952 ± 124
Heat (Kcal/Kg/h) Dark	0.44 ± 0.02	**0.38 ± 0.01***	0.51 ± 0.02	0.48 ± 0.02
Heat (Kcal/Kg/h) Light	0.36 ± 0.02	**0.30 ± 0.01***	0.39 ± 0.02	0.38 ± 0.02
RER (VCO_2/VO_2) Dark	0.87 ± 0.01	0.87 ± 0.005	0.86 ± 0.02	**0.91 ± 0.01$^{\#}$**
RER (VCO_2/VO_2) Light	0.83 ± 0.01	0.83 ± 0.01	0.84 ± 0.02	**0.88 ± 0.01$^{\#}$**
EE Dark	18059 ± 444	**15658 ± 196****	14084 ± 502	14126 ± 665
EE light	14704 ± 722	**12352 ± 410***	12049 ± 929	11543 ± 595

Results represent mean ± standard error. Significant findings are labeled in bold. **CT**, Control (n = 5-7); **EC-Myc KO**, Endothelial c-Myc knockout (n = 8-11). VO_2, Volume of oxygen consumed; VCO_2, Volume of carbon dioxide produced; RER, Respiratory Exchange Ratio; EE, Energy expenditure. *p < 0.05 vs. CT female, **p< 0.01 vs. CT female, #p<0.05 vs. CT male.

Supplementary Table 3 – Echocardiography analysis of control and endothelial c-Myc overexpression mice under control and western diet

MV FLOW	CT-CTD	CT-WD	OE-CTD	OE-WD
A' (mm/s)	-34.32 ± 8.55	-27.16 ± 3.07	-34.69 ± 6.17	-29.75 ± 6.05
E' (mm/s)	-30.45 ± 2.58	-20.57 ± 5.53	-28.02 ± 7.48	-27.44 ± 1.35
MV AET (ms)	51.94 ± 5.28	48.89 ± 3.78	49.72 ± 6.91	52.15 ± 3.88
IVCT (ms)	6.25 ± 1.10	**10.56 ± 1.73***	8.43 ± 1.70	**7.10 ± 1.42#**
IVRT (ms)	14.34 ± 0.94	15.83 ± 3.31	13.70 ± 1.12	12.78 ± 1.88
MV A (mm/s)	511.11 ± 56.83	**209.88 ± 68.19***	382.80 ± 34.31	**427.28 ± 162.99#**
MV E (mm/s)	666.01 ± 85.67	784.93 ± 22.25	804.09 ± 137.85	823.61 ± 75.14
LV MPI	0.39 ± 0.04	**0.54 ± 0.08***	0.45 ± 0.06	**0.38 ± 0.04#**
MV E/A	1.31 ± 0.13	**3.99 ± 1.15***	2.10 ± 0.35	**2.18 ± 0.92#**
MV E/E'	-22.03 ± 3.74	-40.22 ± 11.77	-30.39 ± 11.34	-29.85 ± 2.38
A'/E'	1.11 ± 0.19	1.36 ± 0.22	1.32 ± 0.48	1.08 ± 0.17
E'/A'	0.91 ± 0.16	0.75 ± 0.13	0.84 ± 0.36	0.94 ± 0.17
**LAX (Long Axis)**	**CT-CTD**	**CT-WD**	**OE-CTD**	**OE-WD**
Heart Rate (bpm)	479.04 ± 45.07	473.65 ± 21.14	469.01 ± 67.06	435.85 ± 18.10
Area;s (mm^2)	11.32 ± 2.78	11.12 ± 1.03	11.47 ± 1.45	10.73 ± 2.12
Area;d (mm^2)	18.39 ± 2.62	18.51 ± 0.25	19.13 ± 1.51	18.88 ± 2.10
Volume;s (uL)	18.33 ± 6.83	17.35 ± 2.44	17.89 ± 3.23	16.94 ± 5.91
Volume;d (uL)	42.95 ± 7.92	44.38 ± 1.82	44.87 ± 5.49	44.54 ± 9.72
Stroke Volume (uL)	24.62 ± 4.43	27.04 ± 4.14	26.98 ± 5.41	27.60 ± 4.13
Ejection Fraction (%)	60.51 ± 9.89	60.71 ± 6.82	59.97 ± 7.31	62.70 ± 5.74
Cardiac Output (mL/min)	15.85 ± 5.52	12.82 ± 2.16	12.44 ± 1.62	12.05 ± 1.81
**SAX (Short Axis)**	**CT-CTD**	**CT-WD**	**OE-CTD**	**OE-WD**
Diameter; s (mm)	2.56 ± 0.39	2.34 ± 0.18	**1.94 ± 0.15***	2.24 ± 0.32
Diameter; d (mm)	3.79 ± 0.39	3.65 ± 0.24	3.33 ± 0.17	3.57 ± 0.29
Fractional shortening (%)	32.64 ± 4.16	35.86 ± 1.00	**41.82 ± 1.78***	37.38 ± 4.23
LV mass corrected (mg)	114.12 ± 13.07	**154.71 ± 12.50***	121.57 ± 5.12	**122.27± 7.92#**
LVAW;s (mm)	1.44 ± 0.07	**1.76 ± 0.13***	1.52± 0.05	**1.62 ± 0.07#**
LVAW;d (mm)	1.05 ± 0.02	**1.27 ± 0.06***	0.99 ± 0.10	**1.19 ± 0.03¥**
LVPW;s (mm)	1.30 ± 0.28	1.56 ± 0.04	**1.73 ± 0.18***	**1.43 ± 0.13¥**
LVPW;d (mm)	0.91 ± 0.25	**1.22 ± 0.09***	**1.33 ± 0.12***	**1.00 ± 0.12¥**

Results are expressed as mean ± standard deviation (n = 3-4). Significant findings are labeled in bold. **CT,** Control; **EC-Myc OE,** Endothelial c-Myc Overexpression; **CTD,** Control diet; **WD**, Western diet. **A'**, Velocity at A' Pulse Wave Tissue Doppler; **E'**, Velocity at E' Pulse Wave Tissue Doppler; **MV ET**, Mitral valve Ejection Time; **IVCT**, Isovolumetric Contraction Time; **IVRT**, Isovolumetric Relaxation Time; **MVA**, Pulse Wave Doppler mitral valve atrial peak; **MVE**, Pulse Wave Doppler Mitral valve early peak; **LV MPI**, Left Ventricular Myocardial Performance Index; **MV E/A**, Mitral valve E to A ratio; **MV E/E'**, Mitral valve E velocity to E' velocity ratio; **A'/E'**, Ratio of A' velocity to E' velocity (PW Tissue Doppler Mode); **E'/A'**, Ratio of E' velocity to A' velocity; **Area;s**: Systolic area; **Area;d**, Diastolic area; **Volume;s**, Systolic volume; **Volume;d**, Diastolic volume; **LVAW;S**, Left ventricular anterior wall (systole); **LVAW;d**, Left ventricular anterior wall (diastole); LVPW;s, Left ventricular posterior wall (systole); **LVPW;d**, Left ventricular posterior wall (diastole). ***** p≤0.05 vs CT-CTD; **#** p≤0.05 vs CT-WD; **¥** p≤0.05 vs OE-CTD.

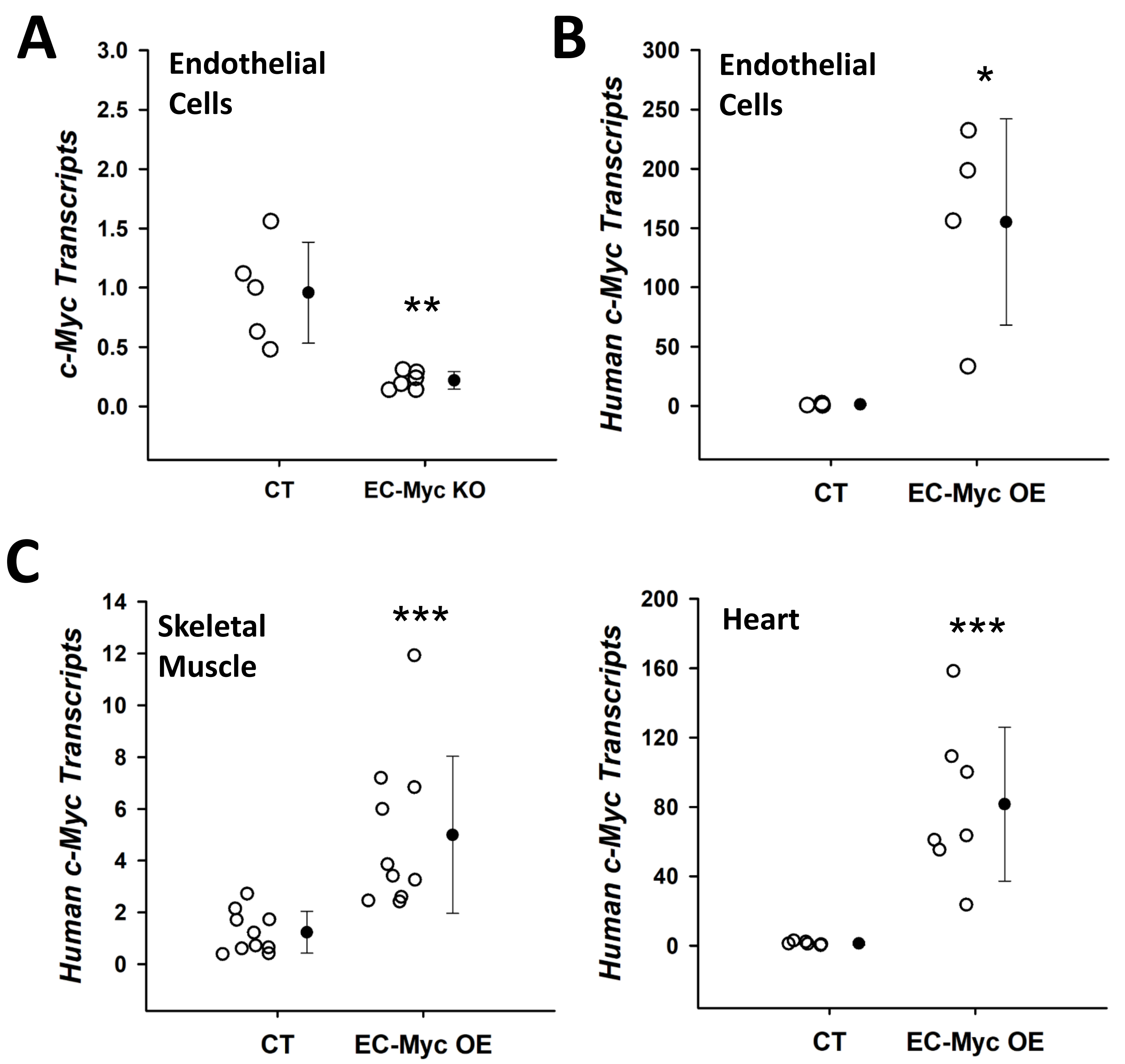

Supplementary Figure 1. Gene expression analysis of c-Myc in tissues and CD31+ sorted cells from endothelial c-Myc knockout and overexpression mice by qPCR. (A) Mouse c-Myc expression in endothelial cells sorted from knockout hearts (n = 5-6). **(B)** Human c-Myc overexpression in endothelial cells sorted from overexpression hearts (n = 4). **(C)** Human c-Myc overexpression in skeletal muscle (n = 10) and heart (n = 7) harvested from overexpression mice. Results are expressed as fold change relative to control. CT, control; EC-Myc KO, endothelial c-Myc knockout; EC-Myc OE, endothelial c-Myc overexpression. *p<0.03; **p<0.003; ***p<0.001.

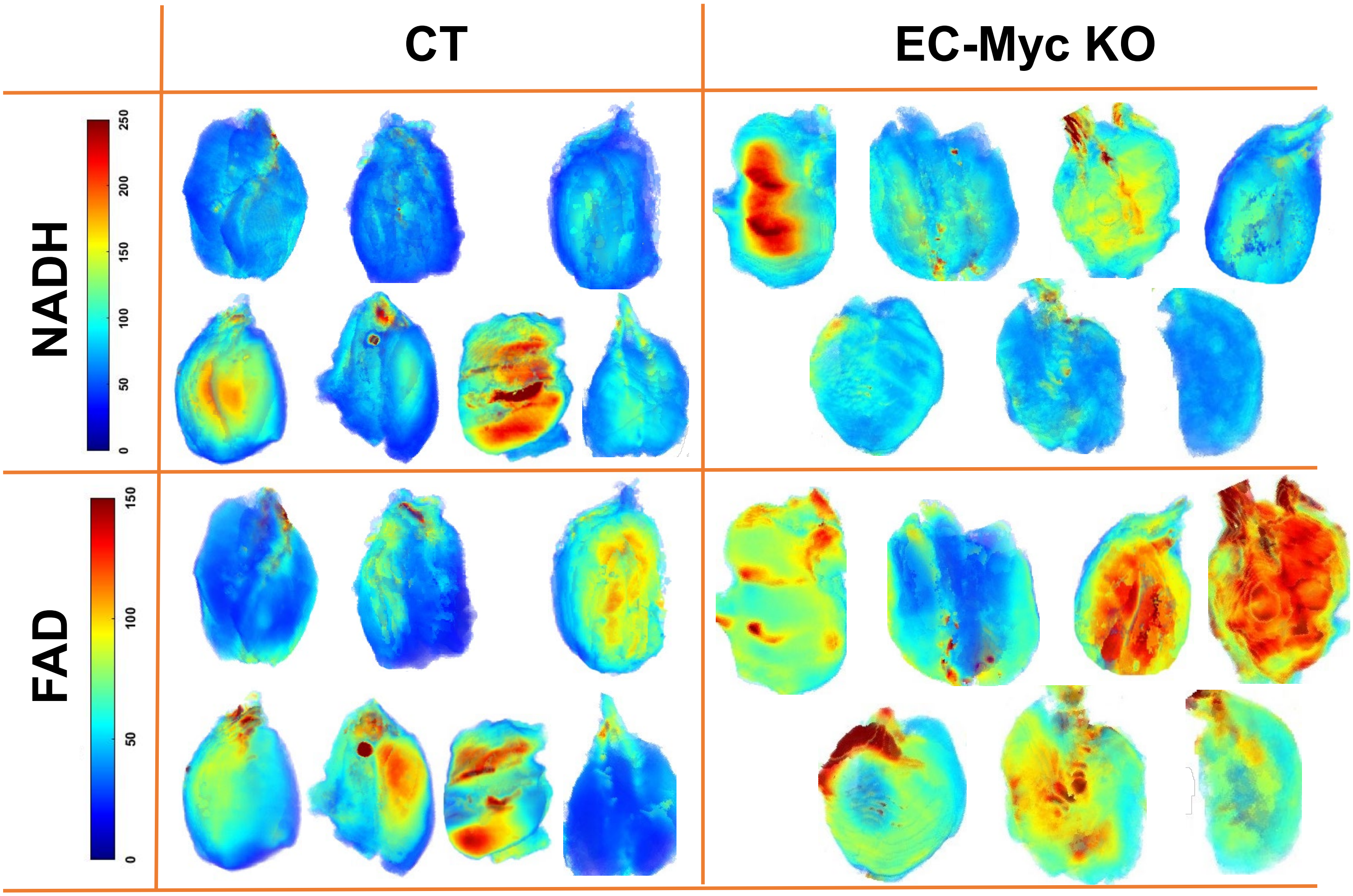

Supplementary Figure 2. Analysis of redox ratio in control and endothelial c-Myc knockout skeletal muscle. (A) Normalized intensities of NADH and FAD images are shown. For each group, seven representative muscle images are presented. CT, Control (n = 7); EC-Myc KO, Endothelial c-Myc knockout (n = 7).

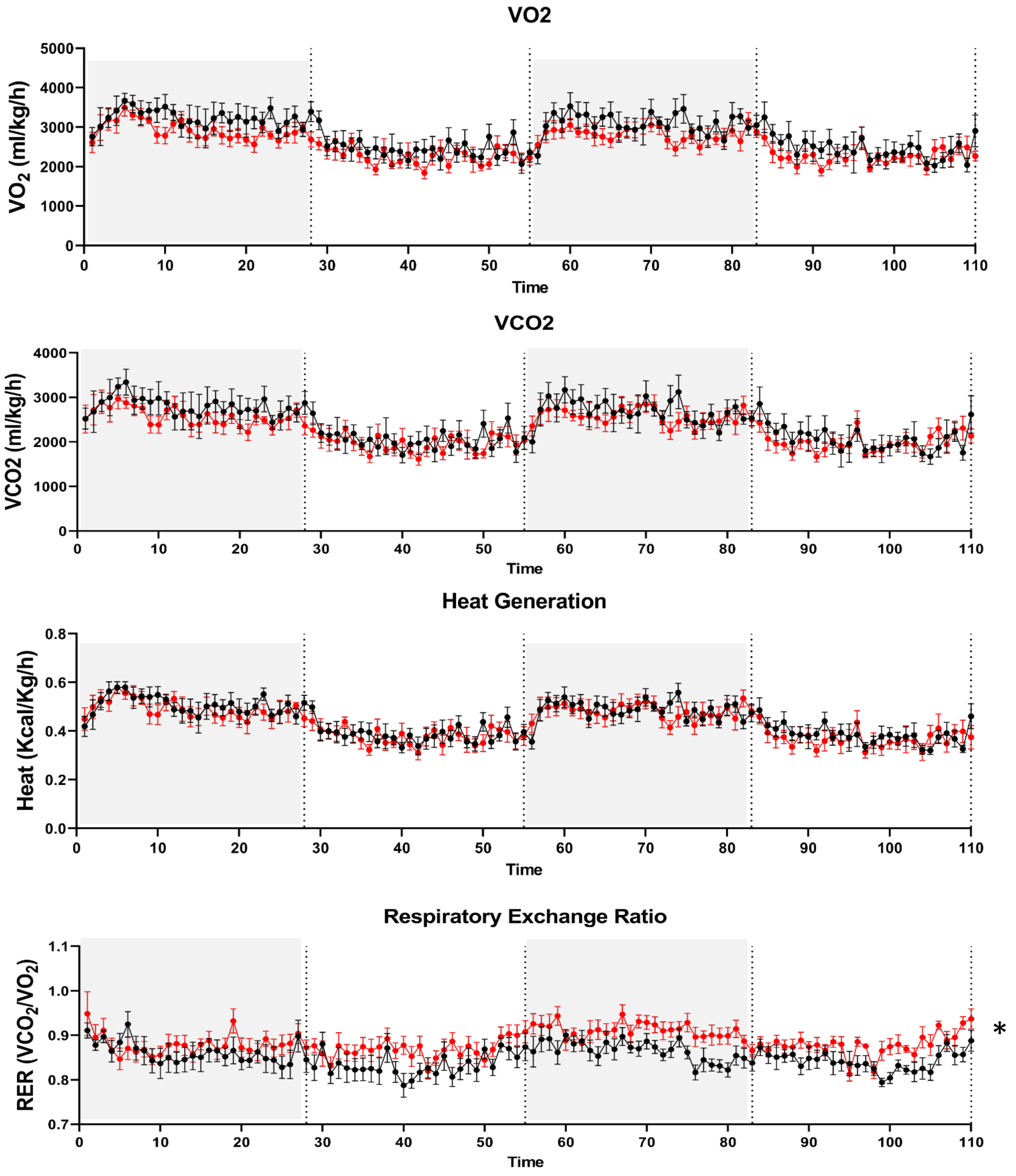

Supplementary Figure 3A. Oxygen consumption (VO_2), Carbon dioxide release (VCO_2), Heat production (Heat) and Respiratory exchange ratio (RER) by indirect calorimetry using the Oxymax Comprehensive Lab Animal Monitoring System (CLAMS) in Control (CT) and Endothelial c-Myc knockout (EC-Myc KO) males. Black lines, CT (n = 7); Red lines, EC-Myc KO (n = 7). Grey, dark; White, light. *$p<0.05$, **$p<0.01$.

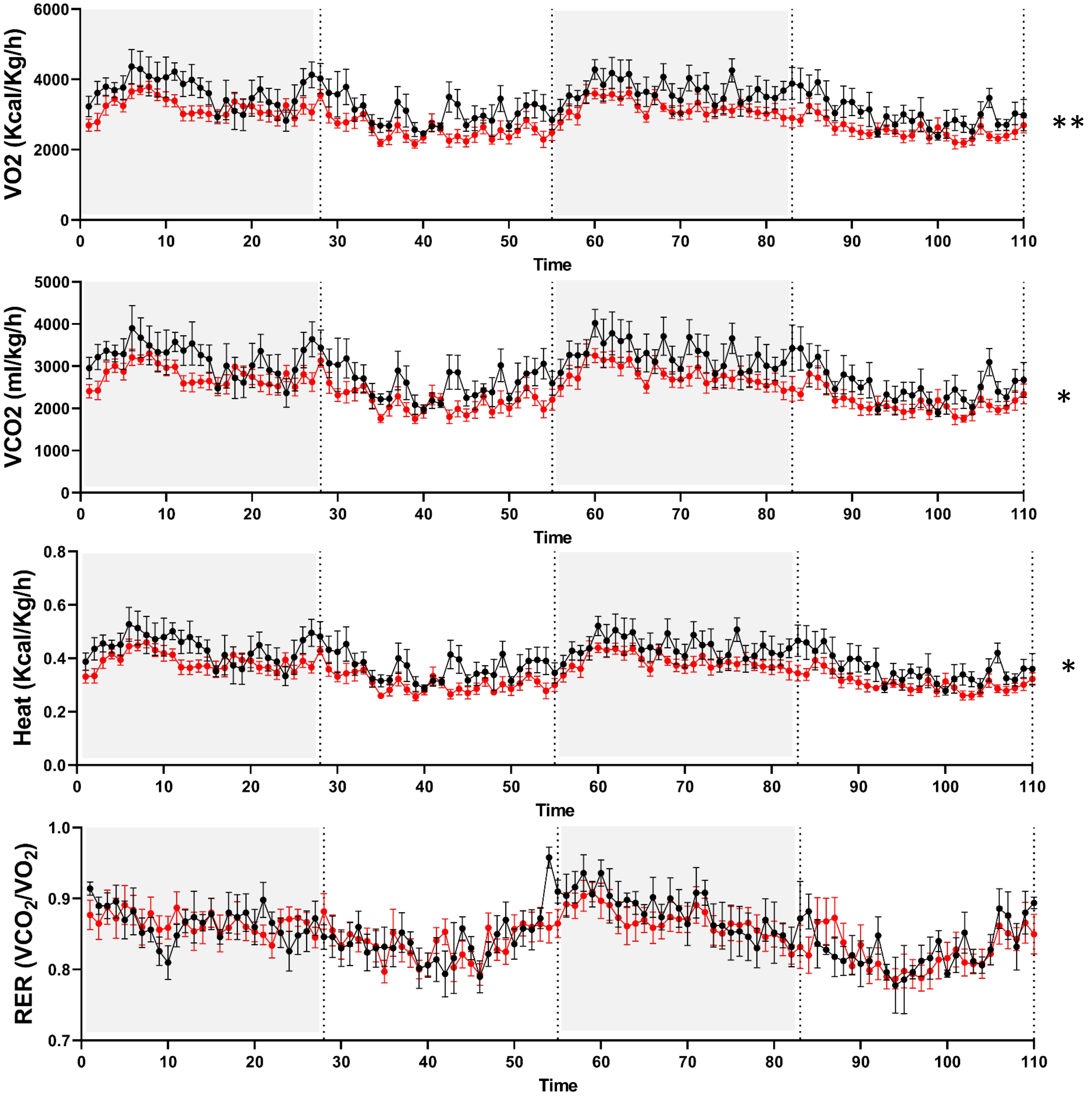

Supplementary Figure 3B. Oxygen consumption (VO_2), Carbon dioxide release (VCO_2), Heat production (Heat) and Respiratory exchange ratio (RER) by indirect calorimetry using the Oxymax Comprehensive Lab Animal Monitoring System (CLAMS) in Control (CT) and Endothelial c-Myc knockout (EC-Myc KO) females. Black lines, CT (n = 5); Red lines, EC-Myc KO (n = 11). Grey, dark; White, light. *p<0.05, **p<0.01.

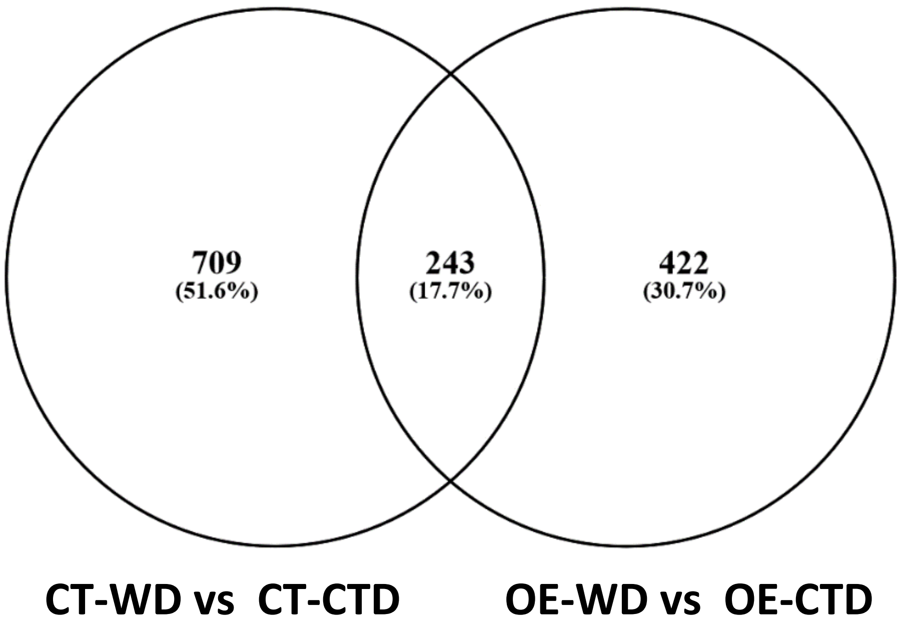

Supplementary Figure 4. Venn diagrams indicating the number of genes significantly altered by western diet exposure in skeletal muscle from control and endothelial c-Myc overexpression mice (n = 3-4). CT, control; OE, endothelial c-Myc overexpression; CTD, Control diet; WD, Western Diet.

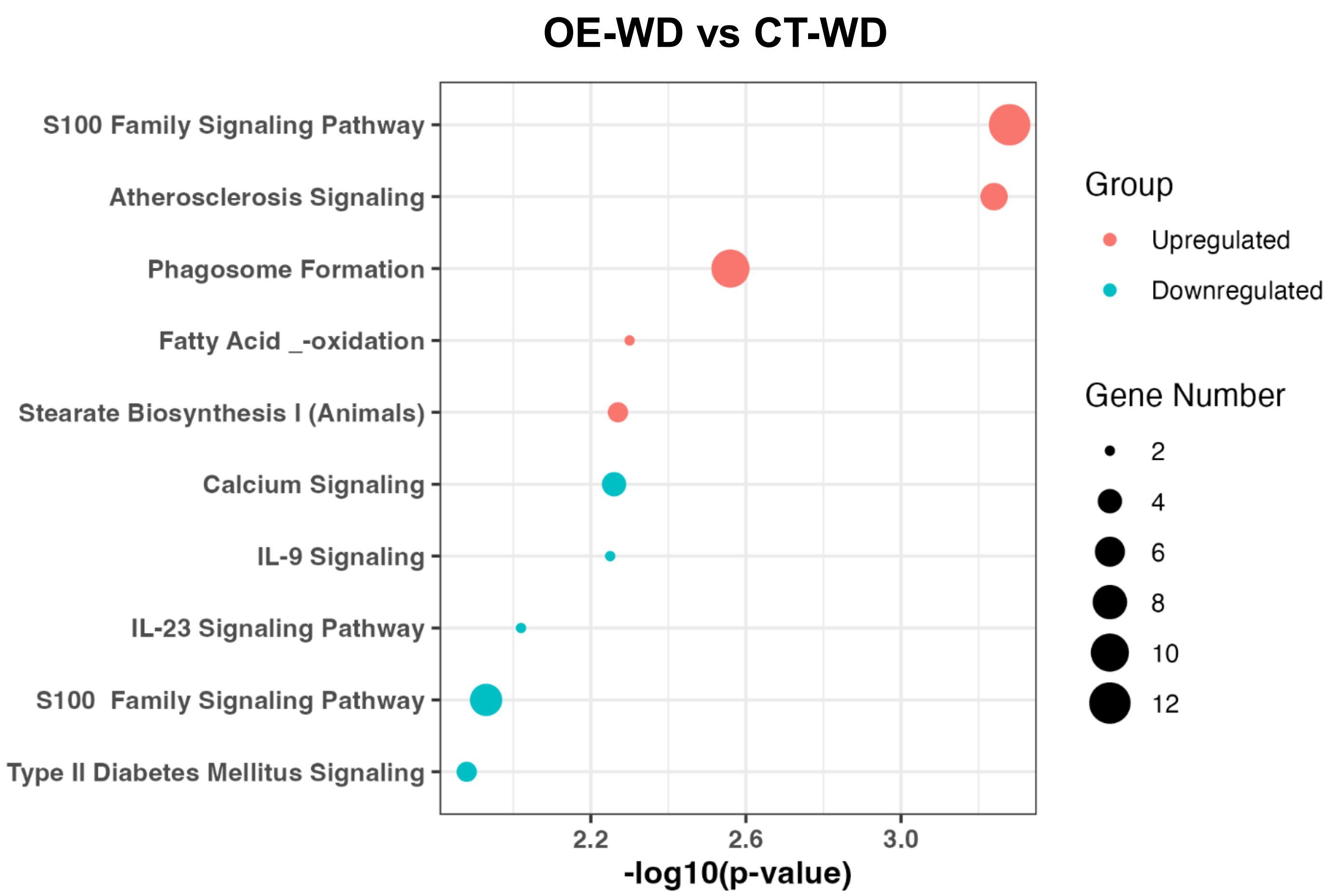

Supplementary Figure 5. Top canonical pathways identified by Ingenuity Pathway Analysis comparing up- and down-regulated genes in endothelial c-Myc OE skeletal muscle relative to CT under western diet. CT, Control; OE, endothelial c-Myc overexpression; WD, western diet. (n = 4).

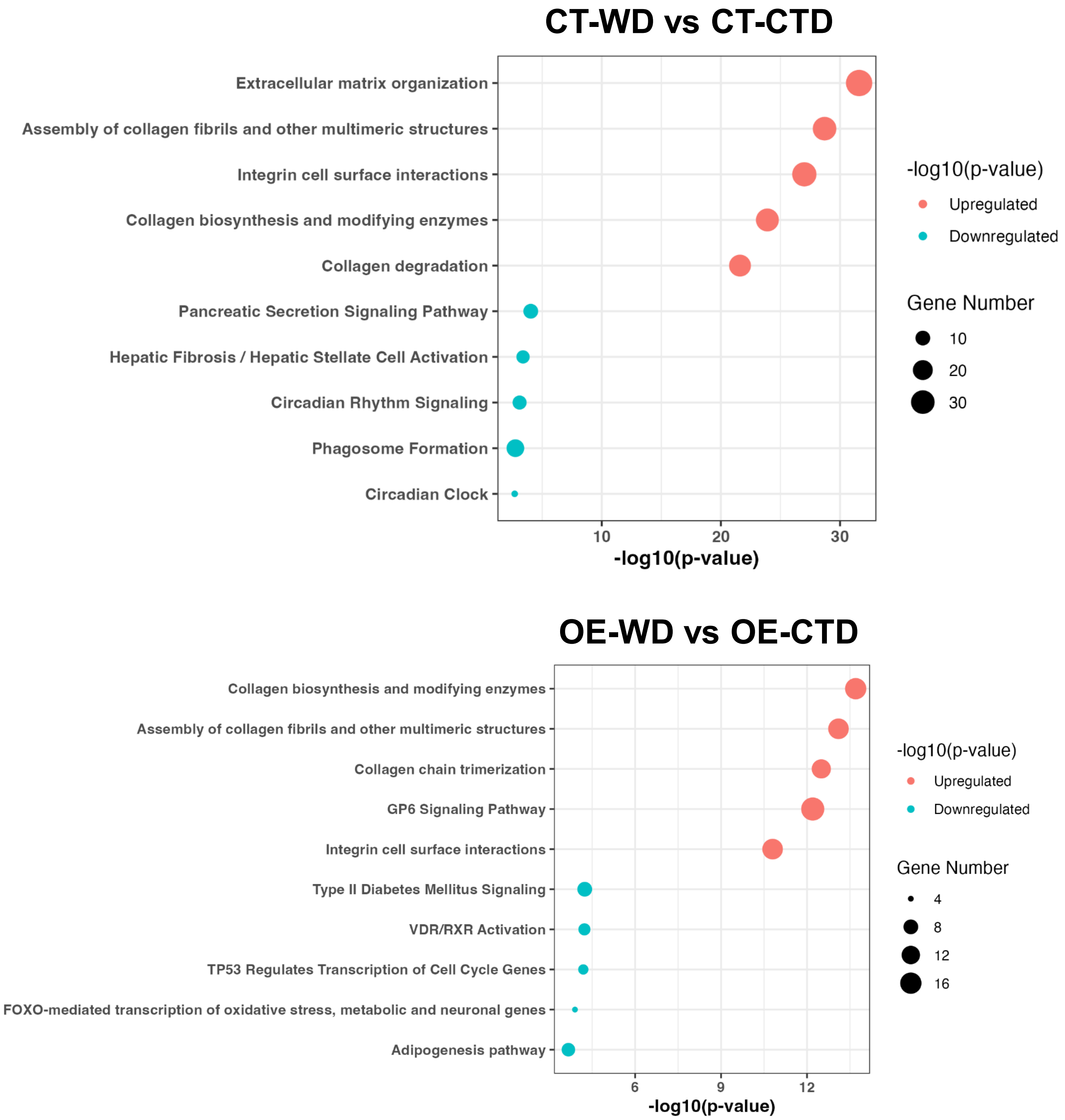

Supplementary Figure 6. Top canonical pathways identified by Ingenuity Pathway Analysis comparing up- and downregulated genes in endothelial c-Myc OE and control skeletal muscle under western diet relative to control diet. CT, Control; OE, endothelial c-Myc overexpression; CTD, control diet; WD, western diet. (n = 3-4).
